# Fijian Farmers' Attitude and Knowledge Towards Antimicrobial Use and Antimicrobial Resistance in Livestock Production Systems–A Qualitative Study

**DOI:** 10.3389/fvets.2022.838457

**Published:** 2022-03-30

**Authors:** Xavier Khan, Rosemary H. M. Lim, Caroline Rymer, Partha Ray

**Affiliations:** ^1^Department of Animal Sciences, School of Agriculture, Policy and Development, University of Reading, Reading, United Kingdom; ^2^Reading School of Pharmacy, School of Chemistry, Food and Pharmacy, University of Reading, Whiteknights, Reading, United Kingdom; ^3^The Nature Conservancy, Arlington, VA, United States

**Keywords:** attitude, knowledge, livestock farmers, antimicrobial use, antimicrobial resistance, Fiji

## Abstract

Antimicrobial resistance (AMR) is a global health issue affecting humans and livestock. To mitigate AMR risks, responsible use of antimicrobials in livestock production systems have been advocated. Studies have reported patterns of antimicrobial use (AMU) in livestock production systems; however, there is limited information on the drivers of AMU and AMR. Therefore, this study aimed to explore and understand the attitude and knowledge of Fijian livestock farmers on AMU and AMR. Livestock farmers and managers from the Central and Western divisions of Viti Levu, Fiji were recruited using purposive and snowball sampling methods. Face-to-face one-to-one semi-structured qualitative interviews were conducted. Interview questions were informed by the Theory of Planned Behavior (TPB). Interview transcripts were analyzed inductively using reflexive thematic analysis and deductively using the TPB framework. A total of 19 cattle and poultry farmers took part. Our analysis generated four themes: (1) Uninformed use of antimicrobials and unaware of AMR, (2) Safeguarding livestock and generating income source as primary motivators for using antimicrobials (3) Medicine shortage results in hoarding and self-prescribing, and (4) Farm decisions on AMU and livestock management influenced by foreign farmers and veterinarians. Livestock farmers used medicines in livestock production; however, they could not differentiate amongst different types of medicine, including antimicrobials. Antimicrobials were used to prevent diseases in livestock and promote production of food and financial security but without any awareness of the risks of AMR. Additionally, farmers hoarded and self-prescribed medicines. Farmers rationed antimicrobials by not completing the entire course of antibiotics to save them for future use. Based on past experiences, farmers expressed dissatisfaction with the veterinary services provided by the government. They sought help online and from foreign farmers and veterinarians. We propose the need for antimicrobial stewardship (AMS) programmes focused on promoting rational use of antimicrobials and awareness of AMR amongst farmers in the Fijian livestock production systems. These programmes need to consider the anthropological, socio-cultural, economic, and environmental factors driving AMU. Future studies are underway to explore the attitude and knowledge of Fijian veterinarians, para-veterinarians and pharmacists on AMU and AMR to gain a broader systems knowledge to inform the design of AMS programmes.

## Introduction

Antimicrobial resistance (AMR) is a global health issue affecting humans and livestock (1, 2). Although the direct links between antimicrobial use (AMU) in livestock production systems and the increase in AMR in humans have yet to be established, the World Health Organization (WHO), World Organization of Animal Health (OIE) and Food and Agricultural Organization of United Nations (FAO) advocate responsible use of antimicrobials across both human and veterinary medicine (1–4).

Livestock farmers use antimicrobials therapeutically; however, there have been concerns that antimicrobials are used prophylactically in herds/flocks of animals without the supervision of a veterinarian (5) and for growth promotion (6, 7) to safeguard livestock production (8), thus maintain food and financial security (9). The European Union (EU) and the United Kingdom (UK) prohibit the use of antimicrobials for growth promotion in livestock production, but this is not the case in other developed and developing countries (7, 10). There are studies reporting patterns of AMU and practice in developing countries (6, 11, 12), but few have explored farmers' behavioral drivers for using antimicrobials in developing countries (13–15), which is key in the design and implementation of antimicrobial stewardship (AMS) programmes (4).

Studies have reported better understanding of AMU and AMR amongst farmers in developed countries compared to developing countries (16, 17). Although some studies have suggested improving farmers knowledge through education optimizes responsible AMU, there is a mismatch between perceived knowledge and understanding, and practice (16–18). Other factors such as farmer's age, years of experience, farm and flock size, and access to veterinary services also influence AMU behavior (16, 17). To date, most studies exploring drivers of AMU and AMR have been conducted in developed and developing countries (16, 17) with very little is known about the Oceania region except for Australia and New Zealand (16, 17, 19). Hence understanding of the drivers of AMU and AMR in the local context is necessary.

Our current study focuses on the livestock farming systems in Fiji. Our recent study demonstrated the considerably high use of antimicrobials in semi-commercial and backyard farming systems in the largest island of Fiji (Viti Levu) (12), but the drivers for AMU in this context remain unexplored. An important step is to understand the livestock farmers' attitude and knowledge, which can shape their AMU behavior (16, 17, 20). Socio-psychological theories such as Theory of Reason Action (TRA) (21), Health Belief Model (HBM) (22) and Theory of Planned Behavior (TPB) (23, 24) have been used as theoretical frameworks to understand and explain people's behavior. In particular, TPB enables understanding of behavior by analyzing people's knowledge, attitude and motivation that affects their decision-making process (24, 25). TPB has been used to understand the behavior of farmers on livestock production and management (14, 15). It has also informed the design and implementation of interventions to promote the prudent use of antimicrobials in farms in Europe (26, 27). There are differences in psychological and contextual drivers (such as legal framework, policies, and procedures) relating to livestock production and management globally (13, 28), therefore the direct application of existing AMS policies may not be effective. Hence, it is imperative to consider the drivers of AMU behavior at the country level to develop interventions promoting the responsible use of antimicrobials (8).

Therefore, this study, informed by TPB, aimed to explore, and understand the attitude and knowledge of Fijian livestock farmers' toward AMU and AMR in the Central and Western division of Viti Levu, Fiji.

## Methods

### Reflexivity and Team

An interdisciplinary research team comprising of two female and two male researchers conducted the study; a male doctoral candidate and pharmacist with experience in agro- security, food security and one health (XK), one female academic pharmacist with a doctoral degree in medicine use and safety and extensive experience in qualitative research (RL), a female animal scientist with a doctoral degree and extensive experience in animal sciences (poultry) (CR) and a male academic veterinarian and animal scientist with a doctoral degree with extensive experience in animal sciences (cattle) (PR). XK undertook all the data collection on the study sites. In preparation, XK undertook qualitative methods research training formally via an accredited course and training 'on the job' with RL and her research team that included XK shadowing another researcher conducting interviews, practical guidance on the analysis of data and mock interviews with RL, CR and PR.

### Study Design and Setting

Face-to-face semi-structured qualitative interviews were conducted between September and November 2019 with Fijian livestock farmers and managers located in the Central and Western divisions of Viti Levu, Fiji. The island of Viti Levu was selected because it is the largest in Fiji, where Fijians lived and raised livestock (29). An interpretivist epistemological position underpinned the design and conduct of our study (30). Reflexive thematic analysis was used as our analytic approach (31, 32). The Consolidated criteria for Reporting Qualitative research (COREQ) was used to report this study (33).

### Participants and Recruitment

Our participants comprised livestock farmers and managers who raised livestock, managed, and directly administered antimicrobials to livestock in their farms. A sample of at least 20 participants from the cattle and poultry production systems (dairy, beef, broiler, and layer) was targeted to generate in-depth, rich accounts and descriptions on AMU and AMR. This study was our follow up study from the earlier published quantitative study which quantified AMU in cattle and poultry production systems (12). Qualitative studies have no ideal sample size (34, 35), and sample sizes of 10–25 have been used in other studies (36, 37). Hence, we presumed 20 participants would be reasonable in our study for generating rich and meaningful insights. Purposive and snowball sampling methods were used to recruit participants. The purposive sampling method allowed diversity in participants and enabled the recruitment of participants who have a direct link, experience and are engaged in the area of interest (38, 39). The participant inclusion criteria are listed in Box 1. The Fijian Ministry of Agriculture livestock officers and assistants working in the major townships in Central and Western divisions identified potential participants and provided participant contact details to XK. XK contacted potential participants via telephone to introduce them to the study. XK visited all participants who were interested in participating in a face-to-face interview. XK provided the participants with the study participant information sheet and obtained verbal informed consent before starting interviews. No participant had any prior relationship with XK.

Box 1 **Participant recruitment criteria.**

• Located in the Central Division or Western Division of Fiji
• From Naitasiri, Namosi, Rewa, Serua, Tailevu, Ba, Nadroga-Navosa or Ra province
• Located on the mainland of Viti Levu.
• Location was accessible by road.
• Over 18 years old age.
• Raised either poultry (layer, meat bird or both) or cattle (dairy, beef, or both) or raised both poultry and cattle (mixed) farms
• Raised livestock in any type of farming systems (subsistence or semi-commercial, or commercial)

### The Interview

TPB informed the development of the initial semi-structured interview guide (24, 40). The semi-structured interview guide was structured around the key constructs of TPB (attitude, subjective norms, and perceived behavioral control) and included structured and probing questions relating to attitude toward treating animals, barriers to treating sick animals, the influence of veterinary professionals and other farmers on farmers and other factors which influenced farmers decisions on using antimicrobials were included. The interview guide was piloted with one participant, and minor changes were made to simplify the questions. See Box 2 for the interview schedule. All interviews were conducted in the English language at a time and location convenient to participants. All participants were encouraged to speak freely and were made aware that XK was interviewing them in the capacity of a PhD researcher.

Box 2 **Interview topic guide.**

1. Can you tell me about your farming experience?

(Prompts: how long farming for? Years of experience in livestock production? Training? Member of any associations?)

2. Can you describe to me a typical working day at your farm?

(Prompts: what do you do? What do you do with your produce?)

3. What do you do when your animals are sick?

(Prompts: Veterinary/ Para-vet consultations? Medicine used? Source? Availability? Cost? Do you record them? How often do you use them? Problems faced?)

4. What do you do when the medicine you use on animals is not working?

(Prompts: consultations? Other farmers? Veterinarians or para-veterinarians? Any other medicine used? How do you use them? Do you follow instructions?)

5. What is your view on why the medicine used did not work?

(Prompts: correct dose? The duration? Right medicine? Type of medicine? Stronger medicine? Didn't follow instructions? Medicine not effective? Antibiotics? antimicrobials?)

6. Can you tell me what is antimicrobial resistance?

(Prompts: if YES: where have you heard from? What do you know about it? What could be done? If NO: Do you think all medicine, you use is antimicrobials? or are they antibiotics? Where did he hear that from?)

7. Are there any other comments you want to make about medicine use or antimicrobial resistance?

### Data Management and Analysis

XK transcribed interview recordings verbatim into MS Word and then checked the accuracy of transcriptions against audio recordings. All interview transcripts were anonymised. The data was analyzed in NVivo 12 (QSR International Pty Ltd., UK) using Braun and Clarke's approach to reflexive thematic analysis; by exploring and establishing patterns in the dataset, emerging topics, and overarching themes (30, 32, 41, 42) (see Supplementary File 1 for an example of the coding of the interview transcripts). The reflexive thematic analytic approach is not underpinned by any theoretical framework, allowing flexibility in the analysis process, leading to the generation of in-depth knowledge on drivers of AMU that a theoretical deductive analytical approach may overshadow (31). To mitigate potential gaps in the analytic approach, data was also analyzed deductively using predetermined topics developed using the TPB framework shown in Box 3 to clarify and compare findings obtained using both approaches. The analysis process was iterative and involved multiple discussions with the research team that also included the clarification of the technical interpretation of the emerging themes in areas of medicine use, poultry, and cattle production. The demographic data were summarized and reported.

Box 3 **Topics.**

• Attitude toward the AMU
• Social influence (AMU subjective norms)
• Perceived behavioral controls of AMU (Perceived behavioral controls)
• Actual behavioral controls

## Results

### Participant Characteristics

A total of 19 livestock farmers and managers participated in the interviews, which lasted between 45 and 50 min (mean 46 min). Table 1 summarizes the demographic characteristics of the participants. Most participants were male (*n* = 13, 68.4%), and 47.4% were 40–59 years of age. Most participants had attained a secondary school education (68.4%, *n* = 13). The majority of the participants were livestock farmers (*n* = 17, 89.5%) and had 0-5 years of experience in livestock farming (*n* = 7, 38.9%). Most participants were dairy farmers (*n* = 6, 31.6%) and raised livestock in semi-commercial farming system (*n* = 12, 63.2%). Over 50% of farms were individually owned (*n* = 10, 52.6%). Most participants had no prior training in livestock production (*n* = 12, 63.2%) and were not members of any association (*n* = 11, 57.9%).

**Table 1:** Demographic characteristics of the livestock farmers (*n* = 19) from Central and Western divisions of Viti Levu^*^, Fiji.

**Category**	**Sub-category**	**N**	**%**
Gender	Female	6	31.6%
	Male	13	68.4%
Age	20–39 years	6	31.6%
	40–59 years	9	47.4%
	Over 60 years	4	21.1%
Division	Central	9	47.4%
	Western	10	52.6%
Province	Rewa	1	5.3%
	Tailevu	2	10.5%
	Naitasiri	3	15.8%
	Namosi	1	5.3%
	Serua	2	10.5%
	Nadroga-Navosa	3	15.8%
	Ba	6	31.6%
	Ra	1	5.3%
Level of education	Secondary	13	68.4%
	Tertiary	4	21.1%
	Vocational agricultural school	2	10.5%
Qualifications	Secondary	13	68.4%
	Tertiary	4	21.1%
	Vocational agricultural	2	10.5%
Occupation	Farmer	17	89.5%
	Farm manager	2	10.5%
Years of experience	0–5 years	7	38.9%
	5–10 years	4	22.2%
	10–20 years	3	16.7%
	Over 20 years	4	22.2%
Enterprise type	Beef	1	5.3%
	Dairy	6	31.6%
	Broiler	3	15.8%
	Layer	2	10.5%
	Broiler and Layer	2	10.5%
	Dairy and Layer	2	10.5%
	Beef and Dairy	1	5.3%
	Broiler and Dairy	1	5.3%
	Beef and Layer	1	5.3%
Farming systems	Backyard	1	5.3%
	Semi-commercial	12	63.2%
	Commercial	6	31.6%
Ownership	Individually owned	10	52.6%
	Family owned (generational)	8	42.1%
	Cooperative owned	1	5.3%
Livestock production training	Yes	7	36.8%
	No	12	63.2%
Association memberships	Yes	8	42.1%
	No	11	57.9%

* *Viti Levu is the largest island in Fiji that is divided into two divisions (Central and Western) and consists of eight provinces listed in the table*.

### Interview Findings

The analysis enabled the generation of four key themes: (1) Uninformed use of antimicrobials and unaware of AMR, (2) Safeguarding livestock and generating income source as the primary motivator for using the antimicrobials, (3) Medicine shortage resulting in the hoarding and self-prescribing, and (4) Farm decisions on AMU and livestock management influenced by foreign farmers and veterinarians.

#### Theme 1: Uninformed Use of Antimicrobials and Unaware of AMR in Livestock

Overall, most participants lacked general understanding and awareness on AMU and AMR and its mechanism of action. Most of the participants did not differentiate between antimicrobials and other types of medicine. They used the terms 'medicine' or 'drugs' to describe any medicine they used, including antimicrobials. They were also unaware of the names of medicines, including antimicrobials, that they were administering to their livestock. Only a few participants knew the names of the disease they treated using the medicines.

“*I don't know the name [of] the medicine; it was the injections. [I] inject them and I don't know what's the name of the medicine” Participant 11*

“*No, we got, um. If we use the, I mean the drugs, there are only two drugs we got, i.e., SA [short acting] and LA [long acting] [penicillin]. Nothing else. And sometimes when they have diarrhea, we give Scourban [and] nothing else” Participant 5*

Most participants referred to medicines by their packaging, the color of the medications or the dosage form of medications instead of their generic or brand names. There were a minority of participants who were aware of the type and class of antimicrobials they used. A few participants described antimicrobials they used as a yellow powder but when probed, they were not able to talk about them further.

“*I used the antibiotic [but I] forgot the name. It [is] some kind of penicillin, I forgot the name written on [that] particular bottle” Participant 7*

“*Yes! [it is] yellow powder, what you use for the chicken. What [do] you call that. I forgot the name of it. It's an antibiotic, we give that”. Participant 2*

According to a few participants, antibiotics were perceived to be a cure for all sicknesses. They used antibiotics on their livestock based on their past experience.

“*Using antibiotics! Um! [Antibiotics] might cure their [sickness or] whatever the [animals] are facing, sickness. yah!” Participant 2*

The participants also shared that they did not know about the antimicrobials and their use in livestock production. But a few participants were able to explain the risks of using the antimicrobials.

“*You have to be quite mindful, [that is] how much you use and [for] how long [you] using for [and] not overusing. So continuous use of antibiotics is harmful to the birds [and] production [as well as] harmful to people. Withholding period has to be maintained”. Participant 2*

Nonetheless, most participants had never heard of AMR and were unable to provide insight into AMR in livestock and its risks. There were a couple of participants, however, who were able to describe their understanding of AMR as linked to a problem in human health.

“*Antibiotic resistance! I don't know about it. We don't have [many cases] of sick birds…”. Participant 6*

“*Yah, I heard of but through human [health]. [I] heard [of it] in humans. In humans, the drugs given to them leads [to] drug resistance. The drugs [are] not effective to their immune system, and it [is] like that eh!” Participant 4*

A few participants highlighted the role of the government to address the risks associated with antimicrobial use.

“*Yeah, they are resistant, but if we just change the medication, then it is ok. If any medication we give every day, it would be like that eh! so we have to change it. If it is harmful, then the government should do something about it”. Participant 14*

#### Theme 2: Safeguarding Livestock and Generating Income Source as the Primary Motivator for Using Antimicrobials

Most participants inherited their livestock farms from their ancestors, and livestock production was their primary source of income. Hence, the sustainability of livestock production was essential to their livelihoods. Mitigating risks on-farm was crucial, and the use of antimicrobials was perceived to be the first line of defense.

A few participants who were contracted milk suppliers expressed confusion with the actions of milk processor companies. These companies rejected their milk products due to the presence of antimicrobial residues even though participants said they had not used antibiotics prophylactically on their livestock during that period. There was therefore a loss of income. To counter further milk rejection by processing companies, one participant treated animals with antimicrobials.

“*My whole weeks' milk was rejected by the [dairy processor]. They said [that] there was antibiotic in the milk and [at] that point in time there were no drugs on the farm that we can [use to] inject for the cows' mastitis. I don't know how this farm had the problem [of] antibiotic [use]. I never [received] any money” Participant 5*

“*I am not sure, but we just give [medicine]. They give the injection for milk if there is milk reject[ion] then they will give the injection, and it will be ok” Participant 14*

Based on past experiences with diseases in their livestock and to mitigate risks introduced from the hatchery and prevent disease transmission on the livestock production, some participants said they will not hesitate to use antimicrobials.

“*Yes! But for the last 2 or 3 years, we never had any issues. If there [are] hatchery issues, we just use Oxytet, and it sorts itself” Participant 13*

“*[Sickness] can be prevented. Sometimes like at the moment, I have some medicine down there for them, for diarrhea [in] young calves, as soon as they get bacteria, I give it to them, and they drink it” Participant 11*

A few participants highlighted that antimicrobial use was necessary due to projected losses resulting from diseases in flocks of chickens from untreated government water supply.

“*When [government water supply] was tested, there was no chlorine in the water. In government water supply, we have E Coli. [When] all [chickens are] toward the end of the batch [cycle that is] Day 28, 29 and 30, if there is no chlorine [in the water] then the E Coli [infects chickens], then we have to use Oxytet, if not, [we will] bear the loss [of income]”. Participant 13*

When using antimicrobials to treat infections in livestock, a few participants said they were selective in the length of treatment. They would monitor the perceived effect of the antimicrobials on their livestock and then act accordingly, whether to continue treatment or to stop.

“*When I see the mastitis [in udder then], I use it. I keep it for, say, about 48 hours, and then I strip it, separate the milk, and I see if [mastitis is] still [present in udder] than I put another one [intramammary unit]. If the milk is [to be] disposed of, then I use the milk. I don't record anything” Participant 10*

The lack of financial security to invest in improving farm infrastructure, conditions, and livestock feed for a few participants led them to use antimicrobials to prevent disease.

”* We used to buy the feed, but it was costly, $38 to $40 [for a bag of feed] and [the] mix of the feed [was not of same quality]” Participant 3*

“*If you properly clean your sheds, then you don't need any medication at all. If your shed is not cleaned properly, then we do have the disease [present in] there “Participant 1*

#### Theme 3: Medicine Shortage Resulting in Hoarding and Self-Prescribing

Many participants highlighted that there was a significant shortage of medicines in Fiji. Therefore, they often buy quantities of medicines, including antimicrobials, which exceed their needs, for future use. The costs of antimicrobials were considered exorbitant, and some participants said they rationed medicines by not completing the course of antimicrobials so that they have some to use in the future. They made these decisions based on perceived response to treatment and the availability of medicines at that time.

“*[For instance,] if the chickens [are] suffering from diarrhea, we just give them for five days, and it's not like we go and buy [only] one packet. We better buy 4 or 5 packets, so one packet [we] use and the rest of the packets we just keep it for future” Participant 17*

“*Yes! It is expensive, and it is not available always. Even now, it's not available. We are unable to afford than what we do is, normally [we use] one tube per teat, [and] if [it is] not available then we use half [tube per] teat, [that is] 50% of tube in one teat and [the remainder]50% in the other [teat]. But [it] depends on the severity of the disease”. Participant 2*

“*Sometimes [antimicrobials are] available, sometimes [it is] not, we just buy all we can and store it. I use [it] when I [need to]” Participant 10*

A few participants expressed that they injected their animals when they suspected any sickness, and based on treatment response, they adjusted the course of treatments.

“*I give it myself. Well, I never experience any of that, but whenever they are sick, I just give that medicine and the problem [is] solved”. Participant 9*

“*I inject them, [and]they do improve when I inject them. Also, I give them Vitamin B complex when they are weak, and they get better” Participant 10*

Other participants said they followed the instructions provided by veterinarians, para-veterinarians, and medicine labels, while the majority expressed that they self-prescribed antimicrobials on farms. A few participants used alternate products such as herbal medicines, electrolytes, feed supplements and kerosene on animals to treat their animals when they do not have access to antimicrobials.

“*I can't because the instructions stated [that] you have to give one per udder, so if I use half, I don't think that it will solve any problem” Participant 12*

“*I [have] used kerosene most of the time [that is] when I don't get the medicine for foot rot”. Participant 10*

#### Theme 4: Farm Decisions on AMU and Livestock Management Influenced by Foreign Farmers and Veterinarians

Most participants did not know that there were livestock associations that they could join to share experiences, access training, and learn about livestock management. They would, however, meet informally with farmers they knew as required, to discuss livestock production and management, including the use of medicines.

“*I haven't had any poultry experience, but I tried [it]myself, like start with only, only chickens and that's how I learnt each day. It's a learning process for me” Participant 9*

“*Yah! my brothers, got a farm further up, and then there are few other farmers who always talk. We always talk if there is an issue on the farm [and] we call each other” Participant 13*

Many participants said that they experienced difficulties accessing their local veterinary services due to the unavailability of professionals. There were often slow to respond to requests for advice from participants. When participants did receive advice from veterinary professionals, they were unsure the information provided could be trusted; the advice given was sometimes perceived to show a lack of experience and knowledge on livestock production and extension services.

“*No! it is very hard. It's no use in calling them because whenever we need them, they are not available there. I don't want to insult anybody, but it does happen that whenever we go to them and try to take advice, they open the book, and they flip the pages. So, it should be when they do a degree, and anything should be at their fingertips. So, they start flipping [the] pages, and they want to tell from there what to do” Participant 1*

“*Service is not good. Sometimes we ring [and they advise] they [will]come tomorrow [however] tomorrow never comes. [I] called [the] veterinarian [and] they never came”. Participant 5*

The majority of participants expressed that due to gaps in the availability of information on livestock production and management locally from veterinary clinics, they sought advice on livestock production and management from farmers and veterinary professionals based in neighboring countries via social media and other communication mediums. Some of these farmers and veterinarians would also visit the participant's farm to provide livestock management related advice.

“*No, it was just on that spot, the same time we got the information from Mr G, and before calling Mr G, I got into google [and] just typed there “what disease [it] is if we notice red spots in the poo of the chicken” same time the disease came about, coccidiosis and the medicine were given there, but I didn't know where to get it [from so] I contacted Mr G in Australia, messaged him, and he told me” Participant 3*

There was also a view from a few participants about the perceived reluctance of some farmers to change their livestock production and management practices.

“*The problem [is] the attitude of the farmers. That's the main thing because farmers can't take advice, so if you are a good farmer, you will take every piece of advice you get and try to implement it. So, Fijian farmers have [an] attitude and [also] the accessibility of information is not enough [for farmers] to access information on (farm management) what to do” Participant 12*

## Discussion

To our knowledge, this is the first study that provided an in-depth insight into the attitude and knowledge of Fijian livestock farmers toward AMU and AMR. Our principal findings were that livestock farmers were uninformed of antimicrobials and were unaware of AMR in livestock. The livestock farmers used medicines to safeguard their livestock, their main source of income to support their livelihoods. Medicine shortage resulted in livestock farmers hoarding medicine, resulting in self-prescribing. Livestock farmers relied on foreign farmers and veterinarians for information and guidance about livestock production, management, and medicine use. They lacked trust in knowledge and advice provided by the local veterinarian and para-veterinarians.

Our findings of livestock farmers lacking knowledge and understanding of AMU and AMR concurs with results demonstrated in studies in other developing countries (43, 44). Low education levels could have led to the lack of knowledge of AMU and AMR (20, 43). However, in our study, the majority of participants had obtained a minimum of secondary school level of qualification (refer to Table 1). Therefore, we believe the lack of awareness and training on medicines in general may have contributed to the lack of knowledge on AMU and AMR amongst participants. The lack of knowledge and understanding of medicine amongst farmers can complicate AMU practice; there are higher chances of incorrect use (45). The lack of knowledge on risks associated with AMU, such as AMR, is of grave concern. A crucial first step in an AMS programme in Fiji would be to include general training and awareness on medicine, including antimicrobials and the risks associated with AMU and AMR to ensure a baseline local knowledge and understanding on medicine use and safety (1, 2). Terminologies and descriptions of the types of medicine need to be demarcated so that livestock farmers and managers have an understanding and be able to differentiate between 'medicine', 'drugs', 'antimicrobials' and 'antibiotics' and not categorize all as 'medicine' or 'drugs' because evidence shows higher chances of incorrect medicine use, including antimicrobials (46).

Participants in our study use antimicrobials in livestock based on their appearance but different medicines may present in similar dosage form, packaging, and color. For instance, intramammary units used for treating mastitis are available in similar dosage forms but used for treating different types of mastitis (dry and wet cow) (47). Similarly, anthelmintic and antibiotic oral powders and solutions are available in the same color with different indications and contraindications (47). Therefore, there is a risk of using medicines inappropriately.

The shortage of medicines can impact overall access and AMU (45, 48). Easy access to antimicrobials and a lack of policies on antimicrobial dispensing (43, 49) have been reported in other countries (18, 50, 51); however, our findings suggest that the medicine shortage in Fiji may have been worsened by livestock farmers hoarding antimicrobials. Additionally, we believe the inconsistent local supply of antimicrobials may have also contributed to a shortage of antimicrobials. However, the hoarding of antimicrobials is of grave concern and have been similarly reported in other studies (52). Farmers who hoard antimicrobials on farms may have better access, therefore, may unsparingly self-prescribe antimicrobials, as reported in other studies, and is a common problem in developing countries (18, 43). We presume the uninformed use of antimicrobials and all other medicine, in general, may also contribute to the shortage of medicine in local veterinary clinics. Our results suggest that farmers with higher socio-economic status, such as semi-commercial and commercial farmers who have a stable income source, may be better positioned to purchase antimicrobials compared to farmers of lower socio-economic status, such as backyard farmers. These backyard farmers are exposed to food and financial insecurity risks and may be unable to treat the animals when needed (9, 53). The hoarding of antimicrobials worsens medicine access, especially when there is a shortage of antimicrobials; therefore, policies promoting rational use of antimicrobials need to be implemented to ensure accessibility and rational use of antimicrobials in livestock production systems (3, 10, 48, 52, 54).

Our results demonstrated that farmers self-prescribed antibiotics and did not complete the full course of antibiotics. The treatment decisions were based on their past experience instead of on the advice of the veterinary professionals, a practice similarly reported in other studies (48). Imprudent use of antimicrobials resulting from self-prescribing with under and overuse have been reported in developing countries (6, 11). Our results also suggest that incomplete courses of antimicrobials took place due to the high costs of the antimicrobials; thus, farmers used antimicrobial sub-therapeutically and saved the rest for future use, which have been similarly reported in other studies (18, 48, 49).

Our findings that antimicrobials were used as the first line of defense prophylactically to prevent the loss of animals and promote production were similar to results demonstrated in studies conducted in other developing countries (18, 43, 49). Some livestock illnesses can result from a lack of nutrition in feeds, which is common in the backyard and semi-commercial farming systems in developing countries where household refuse is used as feeds for livestock (55). Due to the high costs of feeds as compared to medicines, antimicrobials were considered the first line approach to manage illness in livestock for some farmers. Therefore, farmers may self-prescribe antimicrobials to safeguard the livestock and prevent the death of animals that provide food and financial security. The chances of imprudent use of antimicrobials have been shown to be higher when used without consulting veterinary professionals, as reported in other studies (20).

The access and use of antimicrobials have been regulated in many developed countries, which farmers and veterinary professionals mostly adhere to (10); however, the same is not in developing countries (6). Our results suggest the same where antimicrobials were purchased from veterinary clinics, but the actual use of antimicrobials was based on farmers' past experiences, the advice of foreign farmers and veterinarians, other farmers or from an online source. There seemed to be a general lack of confidence in the local veterinary services provided, which concurs with another study (56). Therefore, greater engagement of farmers and veterinary professionals is critically important to regain confidence in the quality of local services provided and to establish a working relationship. When advice is given, there may be other anthropological, socio-cultural, economic, and environmental factors that influence farmers' behavior, as demonstrated in other studies (4, 13, 16, 17, 43, 57). Therefore, these factors need to be taken into consideration when developing AMS programmes.

Overall, our results indicate that local veterinarians and para-veterinarians have little influence on the farm decisions on AMU in the Fijian context. Given that pharmacists are experts in medicine use and safety and are readily accessible in the community, it is surprising that the participants in our study did not consult pharmacists for advice. A study also reported similar findings where antimicrobials were accessed from non-professionals and used without consulting pharmacists (58). The role of the Fijian pharmacist in AMU and AMR is also unknown. Therefore, we suggest further studies exploring the attitude and knowledge of veterinarians and para-veterinarians toward AMU and AMR and studies exploring anthropological, socio-cultural, economic, and environmental factors that may influence AMU behavior in livestock farmers (16, 17). Studies exploring pharmacists' role in AMU and AMR in the livestock production systems are also suggested.

## Limitations and Future Research

This study was the first study that explored the attitude and knowledge of livestock farmers and managers in Fiji toward AMU and AMR. Although our study participants were only concentrated in Viti Levu (29), we consider that our participants provided in-depth insights into the current AMU practice. We acknowledge that the views shared by our participants maybe not be the same as the views of all farmers in Fiji. Due to time and logistical reasons, only the island of Viti Levu was included in this present study. We interpreted and conceptualized participants' accounts, acknowledging that our interpretation may not fully encompass the breadth and depth of their experiences and their attitudes and knowledge of AMU and AMR (30, 31, 41).

TPB was used to explore and understand AMU behavior in this study (14, 15, 17, 59), specifically, to inform the design of the interview guide and analysis of interview transcripts. We acknowledge that there are also limitations to using TPB. TPB does not consider involuntary drivers and the role of emotion (17, 60) as seen in our study where not all themes strongly feature. We therefore also analyzed our data inductively to capture our participants' experiences in-depth. Our study focused on cattle and poultry farmers because it is commonly farmed in Fiji (16, 29). However, future studies need to consider the inclusion of other livestock farmers apart from cattle and poultry in all divisions of Fiji.

## Conclusion

This study provided the first documented accounts of the Fijian livestock farmers attitude and knowledge on AMU and AMR. The study results suggest that there is a lack of knowledge and understanding of AMU and AMR amongst livestock farmers in Fiji. AMS programmes promoting awareness and rational use of antimicrobials and resistance needs to be implemented to increase awareness amongst farmers. These programmes need to consider the anthropological, socio-cultural, economic, and environmental factors driving the irrational medicine use by farmers. Closer collaboration between farmers, veterinarians, para-veterinarians, and pharmacists needs to be forged for successful AMS programmes. Future studies are required to explore the attitude and knowledge of veterinarians, para-veterinarians and pharmacists on AMU and AMR in Fijian livestock production. Lessons learnt may assist in developing additional AMS programmes targeting behavioral interventions.

## Data Availability Statement

The datasets generated for this study will not be made readily available to ensure the confidentiality of participants and may contain potentially identifiable information. The data supporting the conclusions of this study will be available by the authors, upon request, to any qualified researcher. Requests should be made directly to x.r.s.khan@pgr.reading.ac.uk.

## Ethics Statement

The studies involving human participants were reviewed and approved by School of Agriculture Policy and Developments Ethical Committee, University of Reading (Ref #: 00772P). Written informed consent for participation was not required for this study in accordance with the national legislation and the institutional requirements.

## Author Contributions

This study was conceived of and designed by XK, RL, CR, and PR. The interview schedule was drafted, recruitment, interview, recording and transcription, initial coding of transcripts, higher-order analysis and theme development, and drafting of the first manuscript was undertaken by XK. XK, RL, CR, and PR contributed to the development and review. RL, CR, and PR provided comments and revisions through several iterations of the manuscript. All authors contributed to the article and approved the submitted version.

## Conflict of Interest

The authors declare that the research was conducted in the absence of any commercial or financial relationships that could be construed as a potential conflict of interest.

## Publisher's Note

All claims expressed in this article are solely those of the authors and do not necessarily represent those of their affiliated organizations, or those of the publisher, the editors and the reviewers. Any product that may be evaluated in this article, or claim that may be made by its manufacturer, is not guaranteed or endorsed by the publisher.
