# Supplementary material for: Fijian Farmers' Attitude and Knowledge Towards Antimicrobial Use and Antimicrobial Resistance in Livestock Production Systems–A Qualitative Study
Source: Front Vet Sci. 2022 Mar 30;9:838457. doi: 10.3389/fvets.2022.838457 (PMC9007610; doi:10.3389/fvets.2022.838457)
Supplement: Supplementary file 1 [file Table_1.DOCX]

**Supplementary file 1: Coding of semi-structured interview transcripts**

The table below illustrates an extract of the codes and topics for theme.

Different colours were used to represent the codes which lead to the development of the theme. Only codes for the theme is presented in the table.

| **Interview number** | **Transcription** | **Codes** | **Sub-themes** | **Theme** |
| --- | --- | --- | --- | --- |
| Interview 2 | I: Did the veterinarian visit the farm to see the calve?  P: I didn’t go to pick him up and he had improved and there was no need but his coming over tomorrow  I: which medicine you gave to the calve?  P: ummmm…. I did give him medicine, but I only kept him in shade. So, I tried to avoid letting him move to much…so he needs lots of rest only…. get the mother on time let him suck little and let him rest. Let him suck and rest….  I: Do your give any medicine to these chickens?  P: yes! I always have to keep it and give it  I: Which medicine you gave and how did you give it?  P: yah! yes…yellow powder what you call that for the chicken…what you call that. I forgot the name of it…it’s an antibiotic…uh…we give that. | - Aware of names of drugs used, - Aware of name of drugs and indications used, - Aware of dosing - Described medicine by its dosage form and colour - Aware of type of antimicrobial - Unaware of antibiotic resistance - Unaware of name of medicine, - Used medicines on farm, - unaware of AMR, - used antimicrobials for incorrect indications - aware of risks associated with drug resistance - unsure of mechanism of action of AMR - aware of anthelmintic resistance - unclear of mechanism of action of anthelmintic resistance - aware of risks to humans via agri-food chain | - *Knowledge on AMU* - *Risks associated with AMU* - *Knowledge on AMR* | ***Uninformed use of antimicrobials and unaware of AMR in livestock*** |
| Interview 4 | I: Do you know what is antimicrobial resistance?  P: it can be…it can be…. umm.no  I: have you ever heard about antibiotic resistance before?  P: No!  I: have you heard of drug resistance?  P: yah I heard if. But through human…. heard in humans…in humans the drugs given to them their drug resistance ...the drugs not effective to their immune system ...and its like that eh but I haven’t heard in animals.  I: You said earlier that at times you use the medicines for too long and the medicine is not effective. Why do you think like that?  P: I think the bacteria is ……. resistant eh! Other than the drug….  I: what do you think can be done about it?  P: ok just like you get another drug that can fight that you know the bacteria…because it’s just like how we do it in when are dredge the calves… if we keep on using the Nilverm Nilverm Nilverm that Nilverm would be resistant to the ….the bacteria eh…the bacteria that Nilverm would be not useful because the bacteria is like its normal to them for that Nilverm going inside eh…..so that’s how ..we change the Nilverm for certain period than we change …if we see know the…if I see that how I use Nilverm for certain period than now the calves still having known that …the worms….than I better change it ..that’s how I do it. For like 2 years like that …one year …. but I didn’t wait until that time.,. I just use the Nilverm not. When I finish the 20litres and I go and change to another  I: do you have any other comments?  P: lot of antibiotics not good…well because most of the things that we using to animals. same as we using human beings getting from the doctor…the more medicines we use you know it’s not it’s good for your body same as the animals. The more antibiotics you use and it’s not good for the animal health…. rather than if you minimise the use of antibiotics the better to the animal and the better to the human for ...for human consumption that is what I think. |  |  |  |
| Interview 5 | I: do you record all the medicine you use on the farm?  P: no, we got a …. if we use the I mean the drugs there is only two drugs we got SA and LA. nothing else. And sometimes when they have diarrhoea, we give scour ban. nothing else!  I: How do you give Scourban?  P: Yeah! we give three times…10ml….  I: How do you give it .do you follow instructions?  P: Yeah!  I: Do you use any injection on the farm?  P: Only when the big calve gets sick, we inject SA and LA. I remember back 6 months ago 2 of my cows died just because weakness of these dairy officers in Tailevu one died here one died there and another about to die I have to call Dr P. I have to call Dr S. I am so happy that they were here on time, and they save the cow rather than die  I: Do you know what is antimicrobial resistance?  P: .NO  I: Have you have heard about antibiotic resistance?  P: Yeah. about 6 or 7 months later, my whole weeks milk was rejected by FDL and what they said there was antibiotic in the milk and that point of time there was no drug at the farm that we can inject the cow we can the mastitis there was nothing government pharmacy. I mean govt pharmacy or the animal pharmacy there was nothing, but I don’t know it was so 3 4 weeks and it was raining, and all place was swampy I don’t know myself and this farm we got the same problem antibiotic, and they say there is antibiotic here and that come here 4 am and they come and check for days and for one whole week I never get any money. |  |  |  |
| Interview 7 | I: Do give medicines yourself?  P: yes  I: You said that you had an injury on cow, and you used some medicine? Which medicine you used?  P: I used the antibiotic…. I forgot the name…. it’s its…uh…. some kind of penicillin I forgot the name written on particular bottle  I: Did you follow the instruction given by the vet or para-veterinarian?  P: yes. umm sometimes I just use what I have  I: how much you used?  P: I used …. that was 12mls…yah…that’s for a 350kg, 16ml for 400kg  I: did you record it somewhere?  P: no …I didn’t  I: how often did you give that?  P: actually, according to the prescription on the bottle, it supposed to be like today and tomorrow but I. actually antibiotic I didn’t use for healing…. it’s just to so that it could keep going and prevent from. prevent the. prevent the. Fracture from getting worse. So, I applied after 5 days interval. |  |  |  |
| Interview 11 | I: what do you do when the medicine you have given doesn’t work?  P: I call them again.  I: Who do you call and what do they do?  P: they recheck and sometimes I call them about the medicine …doesn’t work for the cows  I: do you use any other medicine?  P: yah!  I: what other medicine you use?  P: I don’t know the name for the medicine .it was the injections …its put in the …inject them and I don’t know what’s the name of the medicine |  |  |  |
| Interview 14 | I: Do you know what is antimicrobial resistance?  P: uh no…if it is harmful, than the the…government should do something about it.  I: what do you think needs to be done by government?  P: it should not be used on the animals so that if it does harm for us, should not use that one.  I: Is there any other comments you want to make?  P: yah. we just need lot of training; farmers should know things like that. |  |  |  |
